# Prognostic Effect of Long Noncoding RNA NEAT1 Expression Depends on p53 Mutation Status in Cancer

**DOI:** 10.1155/2019/4368068

**Published:** 2019-05-02

**Authors:** Masashi Idogawa, Hiroshi Nakase, Yasushi Sasaki, Takashi Tokino

**Affiliations:** ^1^Department of Medical Genome Sciences, Research Institute for Frontier Medicine, Sapporo Medical University School of Medicine, Japan; ^2^Department of Gastroenterology and Hepatology, Sapporo Medical University School of Medicine, Japan; ^3^Biology, Department of Liberal Arts and Sciences Center for Medical Education, Sapporo Medical University, Japan

## Abstract

Recently, many studies have revealed that long noncoding RNAs (lncRNAs) play important roles in various biological and pathological processes. Our previous study reported that lncRNA NEAT1 is a direct transcriptional target of p53. NEAT1 is an essential component of paraspeckles, which have recently been identified as a novel type of nuclear compartment. Although our previous findings indicate that NEAT1 induction contributes to the tumor-suppressor function of p53, the role of NEAT1 in cancer is still controversial. In this study, we comprehensively analyzed the relationship between NEAT1 expression and p53 mutation status. Interestingly, survival analysis based on NEAT1 expression in several cancer tissues revealed that the p53 wild-type group with high NEAT1 expression had a good prognosis, while poor prognosis or no correlation between NEAT1 expression and survival was observed in the p53-mutated group. These results demonstrate that the tumor-suppressive effect of NEAT1 depends on p53 function and is consistent with our previous report showing that NEAT1 supports the tumor-suppressive function of p53. Specifically, NEAT1 seems to play a tumor-suppressive role only in the presence of wild-type p53. These results provide important clues to the roles of NEAT1 in cancer.

## 1. Introduction

p53 is mutated in approximately half of all human cancers and this fact suggests that p53 is one of the most important genes among many tumor suppressors [1]. Furthermore, abnormality of p53 such as mutation or deletion causes poor prognosis and resistance to various cancer therapies [2, 3]. The p53 protein is activated by various cellular stresses such as DNA damage, resulting in transactivation of many target genes related with DNA repair, cell cycle arrest, and apoptosis [4]. Therefore, target genes of p53 have been explored in many studies because p53 executes diverse functions, primarily through transcriptional regulation. So far, we identified several novel p53 target genes that play important roles in p53 function and tumor suppression [5, 6]. Recently, many studies have revealed that long noncoding RNAs (lncRNAs) play important roles in various biological processes and disease mechanisms including cancer [7, 8]. Therefore, we assumed that p53 also transactivates some lncRNAs [9]. Consequently, we found that the lncRNA NEAT1 is a direct transcriptional target of p53 [10]. NEAT1 knockdown had an effect on p53-induced transactivation and enhanced cancer cell growth. Furthermore, low NEAT1 expression was correlated with a poor prognosis in patients with certain types of cancers. These results indicate that NEAT1 induction contributes to the tumor-suppressor function of p53. Around the same time as our findings, Adriaens et al. also identified NEAT1 as a transcriptional target of p53, and they reported the formation of paraspeckles induced by p53 activation [11]. NEAT1 is an essential component of paraspeckles, which have recently been identified as a novel type of nuclear compartment [12–14]. Paraspeckles have important roles in controlling gene expression through retention of RNA containing double-stranded RNA regions [15]. Their results in relation to ovarian cancer indicated that NEAT1 expression is a poor predictor of chemotherapy response. On the other hand, an* in vivo* study revealed that* Neat1* loss provokes global changes in gene expression, resulting in neoplasia promotion [16]. Thus, the NEAT1 role in cancer is controversial, and its precise mechanisms have not yet been elucidated.

In this study, we comprehensively analyzed the relationship between NEAT1 expression and p53 mutation status. Our results provide clues to NEAT1 roles in cancer.

## 2. Materials and Methods

RNA-seq bam files aligned to the human genome (hg38), p53 mutation status determined by whole-exome analysis, and survival information of patients in TCGA (The Cancer Genome Atlas) project were downloaded from the Genomic Data Commons (GDC) portal site (https://portal.gdc.cancer.gov/). Total 11,095 RNA-seq files were analyzed using a series of cufflinks software programs, cuffquant and cuffnorm to quantify and normalize the expression of NEAT1, NEAT1_2 (NR_131012), CDKN1A, and MDM2 genes. The* TP53* gene mutation status in TCGA samples was downloaded from cBioPortal (http://www.cbioportal.org/). Sample numbers are summarized in Supplementary Table 1. Gene expression and prognosis were statistically analyzed using R software.

## 3. Results

**Figure 1:**
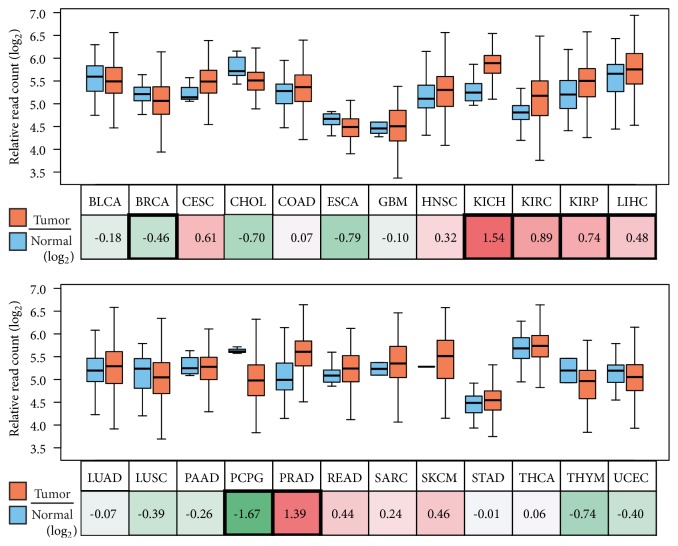
Comparisons of NEAT1 expression between various cancer and normal tissues. RNA-seq data of 24 tumor and normal tissues in TCGA database were analyzed, and NEAT1 expression levels are presented in boxplots. Tumor/normal ratios are indicated as log_2_ values in the box under the tissue labels. Significant changes (p < 0.05 by two-sided Welch's t-test) are marked with a bold frame. Abbreviation for tissue types and sample numbers are summarized in Supplementary Table 1.

In 24 types of human cancer, first, we compared NEAT1 expression between normal and tumor tissues using TCGA datasets (Figure 1, nine cancer tissues were excluded due to no normal sample). In breast invasive carcinoma (BRCA), cholangiocarcinoma (CHOL), and pheochromocytoma and paraganglioma (PCPG), NEAT1 expression was significantly decreased in tumors compared with normal tissues. In esophageal carcinoma (ESCA) and thymoma (THYM), NEAT1 expression was also decreased but without statistical significance. On the other hand, in three renal carcinomas (chromophobe (KICH), clear cell carcinoma (KIRC), and papillary cell carcinoma (KIRP)), hepatocellular carcinoma (LIHC), and prostate adenocarcinoma (PRAD), NEAT1 expression was significantly increased.

**Figure 2:**
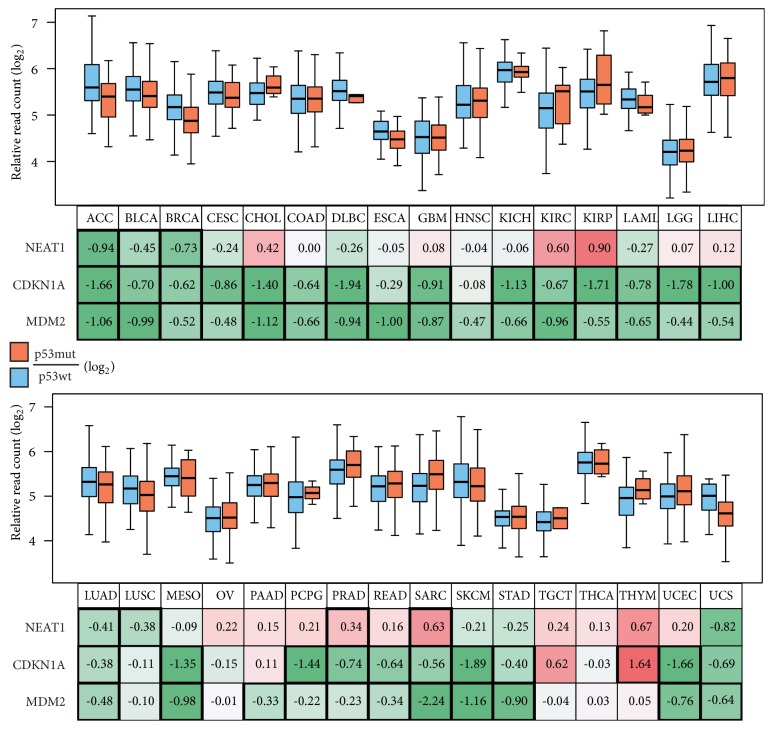
Comparisons of NEAT1 expression between p53 wild-type and mutated cancer types. RNA-seq data of 32 tumor tissues in TCGA database were divided into p53 wild-type (p53wt) and p53 mutant (p53mut) groups and then analyzed, and NEAT1 expression levels are presented in boxplots. p53mut/p53wt ratios are indicated as log_2_ values in the box under the tissue labels. Significant changes (p < 0.05 by two-sided Welch's t-test) are marked with a bold frame. The ratios of other p53 target genes, CDKN1A and MDM2, are also presented (Boxplots for these genes are presented in Supplementary Figure 1). Abbreviation for tissue types and sample numbers are summarized in Supplementary Table 1.

Previously, we found that NEAT1 is a direct transcriptional target of p53 [10]. Therefore, in 32 types of cancer, we tried to analyze the relationship between NEAT1 expression and p53 status (uveal melanoma (UVM) was excluded due to no p53 mutation). RNA-seq data of tumors were divided into two groups, wild-type p53 or mutated p53 (including homologous deletion) groups, and then, the expression of NEAT1 and two major p53 transcriptional targets, CDKN1A (encoding p21) and MDM2, was analyzed and compared among the groups (Figure 2 and Supplementary Figure 1). In adrenocortical carcinoma (ACC), bladder urothelial carcinoma (BLCA), BRCA, lung adenocarcinoma (LUAD), and lung squamous cell carcinoma (LUSC), NEAT1 expression was significantly decreased in p53-mutated tumors compared with p53 wild-type tumors. Uterine carcinosarcoma (UCS) also showed the same tendency without statistical significance. In contrast, NEAT1 expression was significantly increased in p53-mutated PRAD and sarcoma (SARC) tumors; nevertheless, CDKN1A and MDM2 were significantly decreased. The same tendency was observed in CHOL, KIRC, and KIRP (Figure 2).

Human NEAT1 has two isoforms: NEAT1_1 and NEAT1_2. NEAT1_1 is 3.7 kb long, unspliced and polyadenylated. NEAT1_2 is 23 kb long and is also unspliced but not polyadenylated. NEAT1_1 is expressed widely in most human tissues, whereas NEAT1_2 is not abundantly expressed [12, 17]. Although total RNA was treated with oligo dT to select for polyadenylated mRNAs in the RNA-seq protocol used to obtain TCGA data, the NEAT1_2 sequence includes five consecutive T repeats, with at least ten repeats (maximum: 17). Therefore, we speculated that NEAT1_2 is also captured by oligo dT and detected by RNA-seq in TCGA and tried to quantify NEAT1_2 expression in TCGA RNA-seq data. Consequently, we detected significant NEAT1_2 read numbers for analysis in four cancer tissues, ESCA, acute myeloid leukemia (LAML), ovarian serous cyst carcinoma (OV), and stomach adenocarcinoma (STAD) (Supplementary Figure 2). In ESCA and STAD, NEAT1_2 expression was markedly decreased in tumors compared with normal tissues, although the decrease did not reach statistical significance (Supplementary Figure 2A). Furthermore, NEAT1_2 expression was significantly decreased in p53-mutated STAD but not in the other three cancer types (Supplementary Figure 2B).

To examine whether NEAT1 expression affects cancer prognosis, we constructed survival curves using the Kaplan–Meier method and TCGA data (except for testicular germ line tumors (TGCT) due to the low mortality rate, Supplementary Figure 3), and the results are summarized in Table 1. Among 32 cancer tissues, in 11 cancer types (BLCA, BRCA, head and neck squamous cell carcinoma (HNSC), LIHC, LUAD, MESO, OV, rectal adenocarcinoma (READ), SARC, skin cutaneous melanoma (SKCM), and STAD), the rate of survival was significantly higher among patients whose tumors had high levels of NEAT1 expression compared with those with low NEAT1-expressing tumors ("Good" in “Total” column of Table 1). On the other hand, in 7 cancer types, the survival rate was significantly lower among patients whose tumors had high levels of NEAT1 expression than among those with low NEAT1 expression in tumors (colon adenocarcinoma (COAD), glioblastoma multiforme (GBM), KIRC, lower grade glioma (LGG), THYM, uterine corpus endometrial carcinoma (UCEC), and uveal melanoma (UVM)) (“Poor” in “Total” column of Table 1). Thus, the role of NEAT1 may differ in different cancer types.

**Figure 3:**
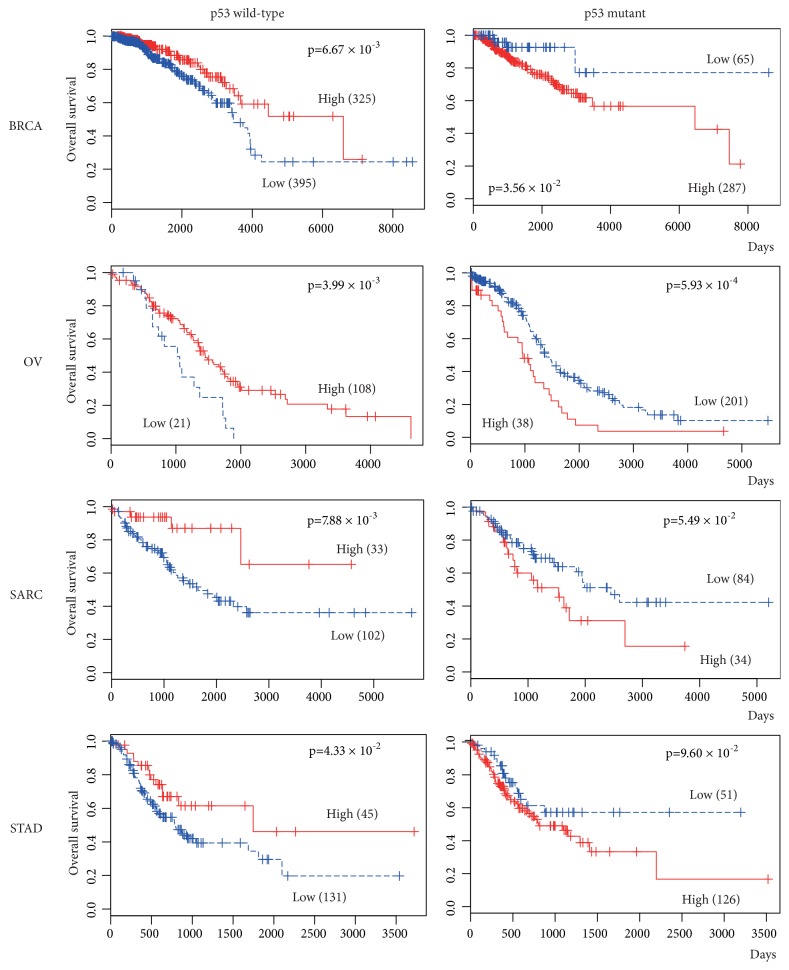
Correlation between NEAT1 expression and prognosis based on p53 status. RNA-seq data of tumor tissues in TCGA database were divided into p53 wild-type and p53 mutant groups, and then, survival was analyzed and plotted using the Kaplan-Meier method. The survival rates for patients with high and low NEAT1 expression are plotted as red and blue lines, respectively. The number of patients in each group is shown in parentheses.* p* values were calculated using a log-rank test.

Our previous report indicated that NEAT1 supports the tumor-suppressive function of p53 [10]. To clarify the p53 dependency of the prognostic effect of NEAT1, tumors were divided into two groups, wild-type p53 and mutated p53 (including homologous deletion) groups, and then, prognosis was analyzed in 18 cancer tissues that had sufficient sample numbers of both wild-type and mutated p53 tumor tissues. Interestingly, in BRCA and OV, the p53 wild-type group with high NEAT1 expression had a good prognosis compared with those with low NEAT1 expression, while poor prognosis was indicated in the p53-mutated group with high NEAT1 expression compared with low NEAT1 expression (Figure 3 and Table 1). The same tendency was observed in SARC and STAD, although the difference in the p53-mutated group did not reach statistical significance (Figure 3 and Table 1). Furthermore, in BLCA, lung squamous cell carcinoma (LUSC), and SKCM, the p53 wild-type group with high NEAT1 expression had a good prognosis compared with the group with low NEAT1 expression, while the p53-mutated groups did not exhibit a significant prognosis difference based on NEAT1 expression (Supplementary Figure 4).

## 4. Discussion

Recently, many papers have been reporting roles of lncRNA NEAT1 in cancer. However, whether NEAT1 has positive or negative effect on human cancer is still controversial. Through gene expression analysis in 24 cancer types using TCGA datasets, we revealed that NEAT1 expression was decreased in five cancer tissues (BRCA, CHOL, PCPG, ESCA, and THYM) and increased in five cancer tissues (KICH, KIRC, KIRP, LIHC, and PRAD) compared with corresponding normal tissues (Figure 1). Thus, a simple comparison of NEAT1 expression between cancer and normal tissues seems to be insufficient to define NEAT1 roles in cancer.

As mentioned in the introduction section, we found that NEAT1 is a transcriptional target of p53. Therefore, as a next step, we sought to confirm whether the expression levels of NEAT1 depend on p53 status. Consequently, six of 32 cancer types (ACC, BLCA, BRCA, LUAD, LUSC, and UCS) indicated a dependency on p53 status for NEAT1 expression (Figure 2). In these cancer types, there would be no room for doubt that NEAT1 expression is strongly regulated by p53. On the other hand, other cancer tissues did not demonstrate a significant difference in NEAT1 expression between p53 wild-type and mutated cancer types; nevertheless, the expression levels of two major p53 target genes, CDKN1A and MDM2, were decreased in p53-mutated tumors (Figure 2). However, this finding does not necessarily mean that NEAT1 expression is not regulated by p53 in these tumors. We consider that the functional ability of p53 to induce NEAT1 expression is preserved in these cancer types although the baseline levels of NEAT1 expression are not significantly affected by p53. In fact, we often observe such a tendency in other p53 target genes. Recently, it was reported that NEAT1 expression and nuclear paraspeckle formation are upregulated by the transcriptional factor HSF1 [18]. Thus, NEAT1 expression may be regulated not only by p53 but also by other transcriptional factors. We also detected a significant level of NEAT1_2 (long form) expression in four cancer tissues (ESCA, LAML, OV, and STAD) (Supplementary Figure 2). Several recent studies have indicated that NEAT1_2 but not NEAT1_1 (short form) is essential for the formation of paraspeckles [13, 14]. In these four cancer types, NEAT1_2 may play functionally important roles.

Furthermore, we performed a survival analysis based on NEAT1 expression. In 11 of 32 cancer types (BLCA, BRCA, HNSC, LIHC, LUAD, MESO, OV, READ, SARC, SKCM, and STAD), high NEAT1 expression suggested a good prognosis but suggested a poor prognosis in seven other cancer types (COAD, GBM, KIRC, LGG, THYM, UCEC, and UVM) (Table 1, Supplementary Figure 3). These conflicting results make it difficult to straightforwardly interpret the NEAT1 role in cancer. Therefore, considering p53 mutational status, we divided tumors into two groups, wild-type p53 and mutated p53 groups, and then, prognosis was analyzed. Surprisingly, in seven cancer types (BLCA, BRCA, LUSC, OV, SARC, SKCM and STAD), the p53 wild-type group with high NEAT1 expression had a good prognosis, while the opposite result or no correlation between NEAT1 expression and survival was observed in the p53-mutated group (Figure 3, Table 1, Supplementary Figure 4). These results support the notion that the tumor-suppressive effect of NEAT1 depends on p53 function and are consistent with our previous report showing that NEAT1 supports the tumor-suppressive function of p53 [10].

## 5. Conclusion

Our results demonstrate that the tumor-suppressive effect of NEAT1 depends on p53 function and is consistent with our previous report showing that NEAT1 supports the tumor-suppressive function of p53. These results provide important clues to the roles of NEAT1 in cancer. Specifically, NEAT1 appears to play a tumor-suppressive role only in the presence of wild-type p53, and NEAT1 may even promote cancer progression in the absence of normal p53 function. To understand the molecular mechanism underlying the prognostic effect of NEAT1, it might necessary to consider p53 status in further studies of NEAT1 function in cancer.

## Figures and Tables

**Table 1:** The relationship between NEAT1 expression and prognosis. Good/Poor: Prognosis of the high NEAT1 expression group compared with the low NEAT1 expression group (p < 0.05 by log-rank test in the Kaplan-Meier method). na: insufficient sample numbers for analysis. p53-status-dependent changes in prognosis are marked with italic font.

		Prognosis in high NEAT1 expression group		
Project	Tissue	Total	p53wt	p53mut
ACC	Adrenocortical carcinoma		na	
BLCA	Bladder urothelial carcinoma	Good	*Good*	
BRCA	Breast invasive carcinoma	Good	*Good*	*Poor*
CESC	Cervical squamous cell carcinoma and endocervical adenocarcinoma			
CHOL	Cholangiocarcinoma		na	
COAD	Colon adenocarcinoma	Poor		Poor
DLBC	Lymphoid neoplasm diffuse large B-cell lymphoma		na	
ESCA	Esophageal carcinoma		na	
GBM	Glioblastoma multiforme	Poor		
HNSC	Head and neck squamous cell carcinoma	Good		Good
KICH	Kidney chromophobe		na	
KIRC	Kidney renal clear cell carcinoma	Poor	na	
KIRP	Kidney renal papillary cell carcinoma		na	
LAML	Acute myeloid leukemia		na	
LGG	Brain lower grade glioma	Poor	Poor	
LIHC	Liver hepatocellular carcinoma	Good	Good	Good
LUAD	Lung adenocarcinoma	Good		
LUSC	Lung squamous cell carcinoma		*Good*	
MESO	Mesothelioma	Good	na	
OV	Ovarian serous cystadenocarcinoma	Good	*Good*	*Poor*
PAAD	Pancreatic adenocarcinoma			
PCPG	Pheochromocytoma and paraganglioma		na	
PRAD	Prostate adenocarcinoma			
READ	Rectum adenocarcinoma	Good	na	
SARC	Sarcoma	Good	*Good*	
SKCM	Skin cutaneous melanoma	Good	*Good*	
STAD	Stomach adenocarcinoma	Good	*Good*	
THCA	Thyroid carcinoma		na	
THYM	Thymoma	Poor	na	
UCEC	Uterine corpus endometrial carcinoma	Poor	Poor	Poor
UCS	Uterine carcinosarcoma		na	
UVM	Uveal melanoma	Poor	na	

## Data Availability

All original data are available in GDC data portal (https://portal.gdc.cancer.gov/) and cBioPortal (http://www.cbioportal.org/). All analyzed data are available from the corresponding authors upon request.

## Supplementary Materials

Supplementary Materials
* Supplementary Figure 1*. Comparisons of CDKN1A and MDM2 expression between p53 wild-type and mutated cancer types.* Supplementary Figure 2*. Comparisons of long form NEAT1 (NEAT1_2) expression.* Supplementary Figure 3*. Correlation between NEAT1 expression and prognosis among cancer patients.* Supplementary Figure 4*. Correlation between NEAT1 expression and prognosis based on p53 status.* Supplementary Table 1*. The summary of analyzed data.
