# Corrigendum to “Prognostic Effect of Long Noncoding RNA NEAT1 Expression Depends on p53 Mutation Status in Cancer”

**DOI:** 10.1155/2019/4757046

**Published:** 2019-08-07

**Authors:** Masashi Idogawa, Hiroshi Nakase, Yasushi Sasaki, Takashi Tokino

**Affiliations:** ^1^Department of Medical Genome Sciences, Research Institute for Frontier Medicine, Sapporo Medical University School of Medicine, Japan; ^2^Department of Gastroenterology and Hepatology, Sapporo Medical University School of Medicine, Japan; ^3^Biology, Department of Liberal Arts and Sciences Center for Medical Education, Sapporo Medical University, Japan

In the article titled “Prognostic Effect of Long Noncoding RNA NEAT1 Expression Depends on p53 Mutation Status in Cancer” [1], there was an error in the Results section. In the third paragraph, the sentence “Although total RNA was treated with oligo dT to select for polyadenylated mRNAs in the RNA-seq protocol used to obtain TCGA data, the NEAT1_2 sequence includes five consecutive T repeats, with at least ten repeats (maximum: 17)” should be corrected to “Although total RNA was treated with oligo dT to select for polyadenylated mRNAs in the RNA-seq protocol used to obtain TCGA data, the NEAT1_2 sequence includes five consecutive A repeats, with at least ten repeats (maximum: 22).”
