# Supplementary material for: Prognostic Effect of Long Noncoding RNA NEAT1 Expression Depends on p53 Mutation Status in Cancer
Source: J Oncol. 2019 May 2;2019:4368068. doi: 10.1155/2019/4368068 (PMC6521466; doi:10.1155/2019/4368068)
Supplement: Supplementary Materials — Supplementary Figure 1. Comparisons of CDKN1A and MDM2 expression between p53 wild-type and mutated cancer types. Supplementary Figure 2. Comparisons of long form NEAT1 (NEAT1_2) expression. Supplementary Figure 3. Correlation between NEAT1 expression and prognosis among cancer patients. Supplementary Figure 4. Correlation between NEAT1 expression and prognosis based on p53 status. Supplementary Table 1. The summary of analyzed data. [file 4368068.f1.zip › Suppl/FigS2.pdf]

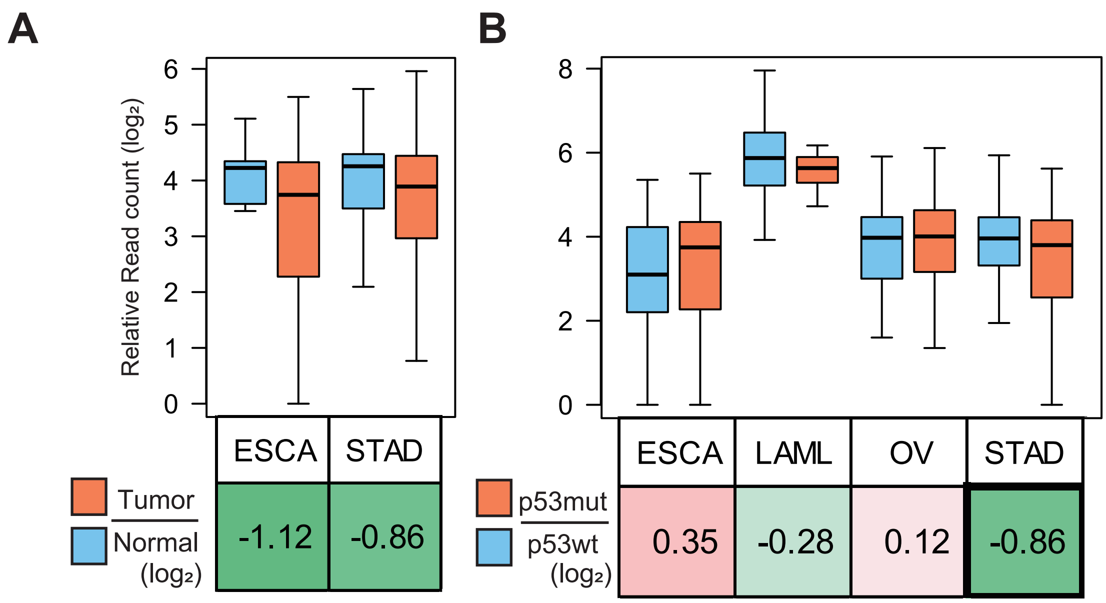


**Supplementary Figure 2.**

Comparisons of long form NEAT1 (NEAT1_2) expression.

(A) RNA-seq data of normal and tumor tissues in TCGA database were analyzed, and NEAT1_2 expression is presented in the boxplot. Tumor/Normal ratios are indicated as log2 values in the box under the tissue labels.

(B) RNA-seq data of tumor tissues in TCGA database were divided into p53 wild-type (p53wt) and p53 mutant (p53mut) groups and then analyzed. NEAT1_2 expression is presented in the boxplot. p53mut/p53wt ratios are indicated as log2 values in the box under the tissue labels.

In (A) and (B), significant changes ($p < 0.05$ by two-sided Welch’ s t-test after the confirmation of normal distributions) are marked with a bold frame.
