# Supplementary material for: Prognostic Effect of Long Noncoding RNA NEAT1 Expression Depends on p53 Mutation Status in Cancer
Source: J Oncol. 2019 May 2;2019:4368068. doi: 10.1155/2019/4368068 (PMC6521466; doi:10.1155/2019/4368068)
Supplement: Supplementary Materials — Supplementary Figure 1. Comparisons of CDKN1A and MDM2 expression between p53 wild-type and mutated cancer types. Supplementary Figure 2. Comparisons of long form NEAT1 (NEAT1_2) expression. Supplementary Figure 3. Correlation between NEAT1 expression and prognosis among cancer patients. Supplementary Figure 4. Correlation between NEAT1 expression and prognosis based on p53 status. Supplementary Table 1. The summary of analyzed data. [file 4368068.f1.zip › Suppl/FigS4.pdf]

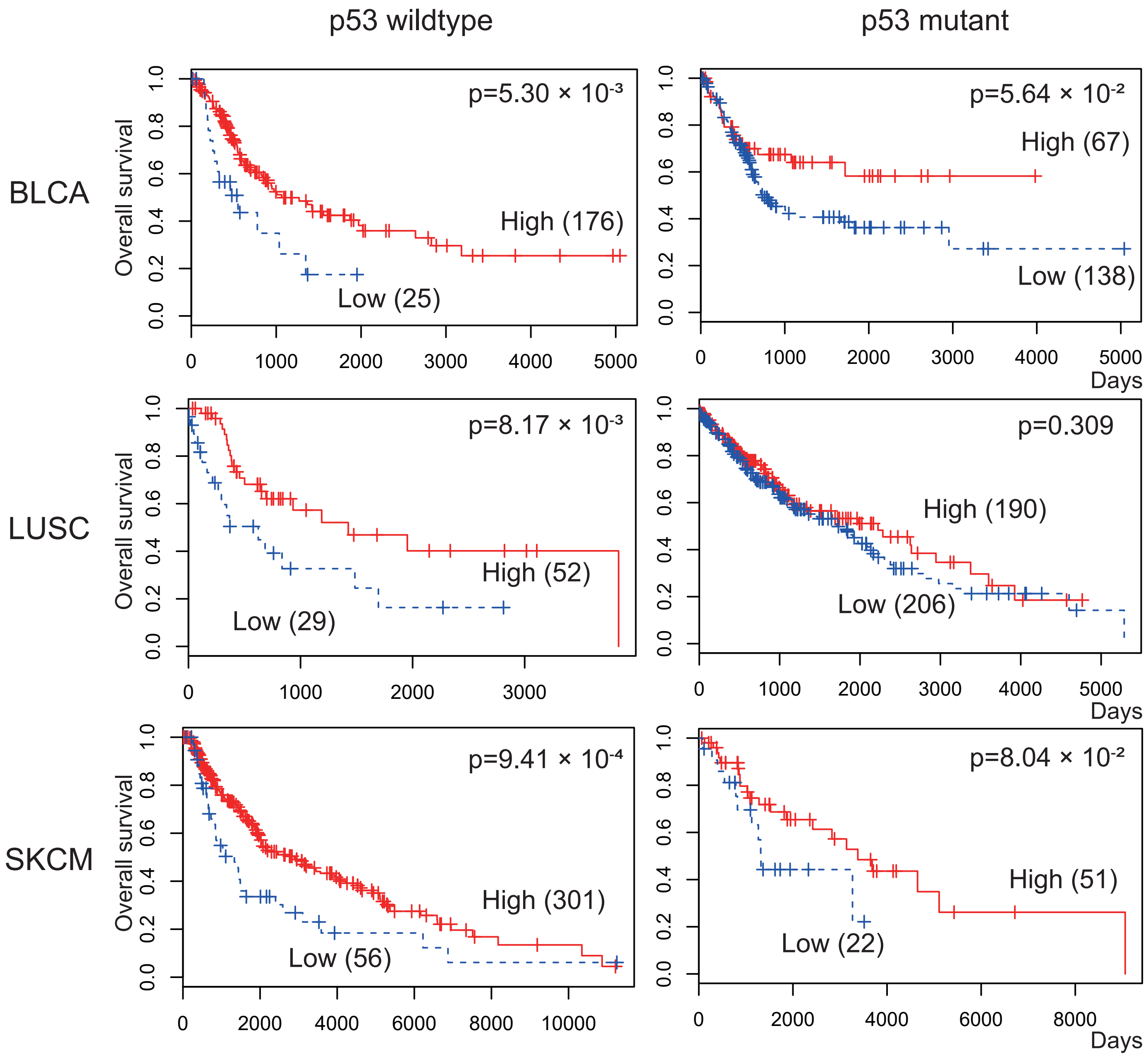

Supplementary Figure 4.

Correlation between NEAT1 expression and prognosis based on p53 status.

Indicated RNA-seq data of tumor tissues in TCGA database were divided into p53 wild-type and p53 mutant groups, and then, survival was analyzed and plotted using the Kaplan-Meier method. The survival rates for patients with high and low NEAT1 expression are plotted as red and blue lines, respectively. The number of patients in each group is shown in parentheses. p values were calculated using a log-rank test.
